# Socioeconomic inequality in barriers for accessing health care among married reproductive aged women in sub-Saharan African countries: a decomposition analysis

**DOI:** 10.1186/s12905-022-01716-y

**Published:** 2022-04-25

**Authors:** Tesfa Sewunet Alamneh, Achamyeleh Birhanu Teshale, Yigizie Yeshaw, Adugnaw Zeleke Alem, Hiwotie Getaneh Ayalew, Alemneh Mekuriaw Liyew, Zemenu Tadesse Tessema, Getayeneh Antehunegn Tesema, Misganaw Gebrie Worku

**Affiliations:** 1 Department of Epidemiology and Biostatistics, Institute of Public Health, College of Medicine and Health Sciences, University of Gondar, Gondar, Ethiopia; 2 Department of Human Anatomy, College of Medicine and Health Science, School of Medicine, University of Gondar, Gondar, Ethiopia; 3 Department of Physiology, School of Medicine, College of Medicine and Health Sciences, University of Gondar, Gondar, Ethiopia; 4 Department of Midwifery, School of Nursing and Midwifery, College of Medicine and Health Sciences, Wollo University, Dessie, Ethiopia

**Keywords:** Socioeconomic related inequality, Erreygers Concentration Index, DHS, Decomposition analysis, Barriers for accessing health care, Sub-Saharan Africa

## Abstract

**Background:**

Accessibility of health care is an essential for promoting healthy life, preventing diseases and deaths, and enhancing health equity for all. Barriers in accessing health care among reproductive-age women creates the first and the third delay for maternal mortality and leads to the occurrence of preventable complications related to pregnancy and childbirth. Studies revealed that barriers for accessing health care are concentrated among individuals with poor socioeconomic status which creates health inequality despite many international organizations top priority is enhancing universal health coverage. Therefore, this study aimed to assess the presence of socioeconomic inequality in barriers for accessing health care and its contributors in Sub-Saharan African countries.

**Methods:**

The most recent DHS data of 33 sub-Saharan African countries from 2010 to 2020 were used. A total sample of 278,501 married reproductive aged were included in the study. Erreygers normalized concentration index (ECI) and its concentration curve were used while assessing the socioeconomic-related inequality in barriers for accessing health care. A decomposition analysis was performed to identify factors contributing for the socioeconomic-related inequality.

**Results:**

The weighted Erreygers normalized Concentration Index (ECI) for barriers in accessing health care was − 0.289 with Standard error = 0.005 (*P* value < 0.0001); indicating that barriers in accessing health care was disproportionately concentrated among the poor. The decomposition analysis revealed that wealth index (42.58%), place of residency (36.42%), husband educational level (5.98%), women educational level (6.34%), and mass media exposure (3.07%) were the major contributors for the pro-poor socioeconomic inequalities in barriers for accessing health care.

**Conclusion:**

In this study, there is a pro-poor inequality in barriers for accessing health care. There is a need to intensify programs that improve wealth status, education level of the population, and mass media coverage to tackle the barriers for accessing health care among the poor.

## Background

Health related inequality is a systematic difference in health across an individuals or according to socially relevant groupings such as between more and less advantaged groups [1]. Globally, it becomes one of the key challenges for public health [2]. In late 2015, Sustainable Development Goal (SDG) were launched to address universal health coverage that aimed to ensure healthy lives and promoting well-being for all at all ages [3, 4]. However, socioeconomic inequalities in health and health-related outcomes are particularly common in low and middle income countries like Sub-Saharan African countries where the poor are disproportionately affected [5, 6].

This inequality becomes much prominent in accessing health care which is defined based on availability, affordability, accessibility, and acceptability of health services [7]. Accessibility of health care is an essential for promoting healthy life and preventing diseases, disabilities and premature deaths, and enhancing health equity for all [8, 9]. Besides, maternal and child health status and equitable distribution health services are the key indicators of the country’s socio-economic status and community [10, 11]. As a result, addressing health inequality has become a top priority for international organizations [12–14]. Despite the Sustainable Development Goals (i.e., Goal 10) set by the United Nations aims to reduce inequality within and among countries [15], maternal health problems are still a major concern and primary agendas for low and middle-income countries like countries in Sub-Saharan Africa (SSA) that contains 19 countries having higher maternal mortality ratio from 20 countries with high maternal mortality ratio in the world [16].

Previous studies have documented that barriers for accessing health care among women was affected by socioeconomic and demographic, cultural characteristics, and geographical disparity [17–23]. Previously the three delays model was developed to evaluate the condition for maternal mortality. These are delay in deciding to seek care, delay in reaching a healthcare facility, and delay in receiving care at the healthcare facility. The presence of Barriers in accessing health care among reproductive-age women creates the first and second delay for maternal mortality [24] and leads the occurrence of preventable complications related to pregnancy and childbirth that includes hemorrhage, infections, hypertension disorders of pregnancy, obstructed labor, unsafe abortion, and can also end up with maternal and child death [9, 23, 25, 26].

Although reducing inequality is the current central objective of health policy in many countries, progress has been inadequate particularly in SSA countries [19]. In spite of the above-mentioned studies conducted on barriers on health care access, up to our knowledge, there is no study conducted to assess the socioeconomic related inequality in barriers for accessing health care in the world, particularly in SSA. Therefore, this study aimed to assess the presence of socioeconomic inequality in barriers for accessing health care and its contributors in sub-Saharan African countries using the recent demographic and health surveys using decomposition analysis. This will help countries to track their progress towards the SDG and ensure their disadvantaged or hard-to-reach populations are not left behind [27, 28].

## Methods

### Data source and sampling procedure

The most recent sub-Saharan African Countries Demographic and Health Surveys (DHS) data conducted from 2010 to 2020 was used for this study. This study analyzed a multi-country DHS dataset that is collected every 5-year across low-and middle-income countries because the program uses standardized tools and follows similar procedure. The DHS program employs two-stage stratified cluster sampling technique where clusters/enumeration areas (EAs) were randomly selected from the sampling frame (i.e. are usually developed from the available latest national census) in the first stage. In the second stage, systematic random sampling was employed to select households in each cluster or EA. Finally, interviews were conducted from the selected households with target populations that are women aged 15–49 and men aged 15–64. In this study, a total weighted sample of 278,501 married reproductive aged women who had given birth within the 5 years preceding the survey of each country were included. In addition, the reproductive aged women with missing value of the outcome variable were excluded from the study (Table 1).

**Table 1:** Overall sample size and sample per each country DHS and survey year

Country	Survey year	Weighted sample size
Angola	2015/16	7957
Burkina Faso	2010	13,555
Benin	2017/18	11,169
Burundi	2016/17	9782
Central democratic Congo	2013/14	12,085
Congo	211/12	6271
Cote d’vore	2011/12	6291
Cameroon	2018	7749
Ethiopia	2016	10,224
Gabon	2012	4443
Ghana	2014	5321
Gambia	2019/20	5321
Guinea	2018	7526
Kenya	2014	7728
Comoros	2012	3218
Liberia	2019/20	4216
Lesotho	2014	3613
Mali	2018	8568
Malawi	2015–16	16,131
Mozambique	2011	9332
Nigeria	2018	29,090
Niger	2012	9868
Namibia	2013	3116
Rwanda	2014/15	6978
Sera lone	2019	9715
Senegal	2010	10,346
Chad	2014/15	4560
Togo	2013	6267
Tanzania	2015/16	8211
Uganda	2016	11,224
South Africa	2016	1461
Zambia	2018/19	7649
Zimbabwe	2015	6152

### Measurement of variables

Socioeconomic-related inequality in barriers for accessing health care was the outcome variable in this study. Barriers for accessing health care were composite variable from four questions related to challenge for health care access (obtaining money, distance to health facilities, permission to consult the doctor, and not wanting to go alone). If women reported at least one challenge of the health care access were considered as having barriers for accessing health care while if a woman didn’t report none of the above challenges were considered as no barriers for accessing health care [29]. The socioeconomic-related inequality of barriers for accessing health care was expressed as the covariance between barriers for accessing health care and the measurement for socioeconomic class which was wealth index in our case. Then, it was classified into either pro-poor, pro-rich, or no inequality.

Women’s age, educational level, wealth index, sex of household head, mass media exposure, place of residence, husbands educational level, current working status, parity, ownership of the assets, women involvements on decision making [30] were incorporated as explanatory variables. The socioeconomic status was measured using the wealth index from DHS data sets. In the DHS data, the wealth index was constructed using principal component analysis for urban and rural separately and then categorized as poorest (quintile 1), poorer (quintile 2), middle (quintile 3), richer (quintile 4), richest (quintile 5) [13, 31–33].

### Data management and statistical analysis

Data were managed and analyzed using STATA 14 software according to the DHS guideline. Sampling weight was considered to adjust for the unequal probability of selection of the sample and the possible differences in response rates. The frequency and different summary measures were used. Pearson’s chi-squared test with its *P* values was reported to indicate the distribution of respondents’ background characteristics.

A concentration index (CI) was computed to measure the socioeconomic-related inequality in barriers for accessing healthcare. For an unbound variable, the concentration index ranges between − 1 and 1, and for unbounded variables, it ranges from μ − 1 to 1 − μ [34]. Decomposition of the healthcare inequality depends on the assumption that the health variable is a linear function of the explanatory variables. Our health variable is a barrier for accessing health care is a binary variable which ranges from 0 to 1 and can’t be negative. Therefore, we used Erreygers normalized concentration index (ECI) which is a modified version of the concentration index was computed [35]. Mathematically, ECI can be defined as:

$${\text{ECI}} = 4*\upmu *{\text{CI(y)}}.$$

where ECI is Erreygers concentration index, CI(y) is the generalized concentration index and μ is the mean of the health variable, barriers for accessing healthcare. Then, the ECI with the standard error (SE) was reported in this study.

To graphically depict the socioeconomic related inequality in barriers for accessing health care, Concentration curves were used and the curves demonstrate the cumulative share of barriers for accessing health care on the y-axis against and the cumulative share of women ranked by the wealth index on the x-axis, arranged from the poorest to the richest. The ECI will be zero in the case when there is no socioeconomic-related inequality. This means if everyone, regardless of wealth status, has the same condition for accessing health care, the concentration curve lies at a 45-degree line (the line of perfect equality).

When the curve lies above the line of equality (when the ECI takes a negative value) the health variable in this case barrier is concentrated among the poor (pro-poor). However, the ECI value can be positive, the curve will be below the line of equality indicating the health variable is concentrated among the rich (pro-rich) [13, 36]. Visual inspection of a concentration curve can give information regarding whether the concentration curve lies above or below the line of equality. To assess the statistical significance of the difference between the concentration curve and the line of perfect equality (45-degree or diagonal line), the ECI with its p-value was calculated.

To identify the relative contribution of various factors to the socioeconomic-related inequality in barriers for accessing health care, decomposition of the ECI was performed [13, 34, 36]. For any linear additive regression model of health outcome (y) [13],

$$y=\mu +\sum_{k}{\beta }_{k}{X}_{k}+\in$$

The concentration index for y, CI, is given as:

$$y=\sum _{k}\left(\frac{{\beta }_{k}{\overline{X} }_{k}}{\mu }\right){C}_{k}+\frac{{gc}_{\in }}{\mu }$$

where “y” is the health outcome variable (in this case socioeconomic related inequality of barriers for accessing health care), ${X}_{k}$ is a set of the socioeconomic determinants of the health outcome, α is the intercept, ${\beta }_{k}$ is the coefficient of ${X}_{k}$, µ is the mean of y, ${\overline{X} }_{k}$ is the mean of ${X}_{k}$, ${C}_{k}$ is the CI for ${X}_{k}$, ${gc}_{\in }$ is the generalized CI for the error term ($\in$), $\frac{{\beta }_{k}{\overline{X} }_{k}}{\mu }$ is the elasticity of y with respect to ${\overline{X} }_{k}$ [34, 37].

### Ethical consideration

This study is a data from the DHS program, so it does not require ethical approval. However, online registration and request for measure DHS were conducted for accessing the data. The dataset was downloaded from DHS on-line archive (http://www.dhsprogram.com) after getting permission. All methods were carried out in accordance with the Declaration of Helsinki.

## Result

### Background characteristics of the study participants

A total of 278,501 weighted married reproductive aged women were included in the analysis. Of those, 181,133 (65.04%) were rural dwellers. Less than one in four, 65,472 (23.51%), of the study participants had ownership on their asset. Moreover, 113,323 (40.69%) of the women were participated in decision making. Regarding barriers for accessing health care, nearly two third, 63.58% (63.40, 63.76) of married reproductive aged women in SSA had barriers for accessing health care. More than three in fourth of women who had barriers for accessing health care were from the poorest households and 71.89% of women having barriers for accessing health care had no formal education (Table 2).

**Table 2:** The weighted proportion of barriers for accessing health care among married women in sub-Saharan Africa by background characteristics of the study participants

Variables	Category	Barriers for health care access		*P* value
		No	Yes	
Regions of SSA	Central	10,390 (24.13)	32,674 (75.87)	*P* < 0.0001
	East	36,112 (37.00)	61,483 (63.00)	
	North	4783 (58.41)	3406 (41.59)	
	West	50,141 (38.67)	79,515 (61.33)	
Age	15–19	6609 (35.01)	12,268 (64.99)	*P* < 0.0001
	20–24	17,305 (36.07)	30,671 (63.93)	
	25–29	22,381(37.39)	37,475 (62.61)	
	30–34	9510 (37.49)	32,524 (62.51)	
	35–39	16,230 (36.63)	28,077 (63.37)	
	40–44	11,168 (35.60)	20,201 (64.40)	
	45–49	8224 (65.86)	15,863 (34.14)	
Residence	Urban	48,710 (50.03)	48,658 (49.97)	*P* < 0.0001
	Rural	52,715 (29.10)	128,419 (70.90)	
Educational level	No education	31,891 (28.11)	81,577 (71.89)	*P* < 0.0001
	Primary	29,935 (33.95)	58,227 (66.05)	
	Secondary	31,606 (48.42)	33,663 (51.58)	
	Higher	7995 (68.89)	3610 (31.11)	
Sex of household head	Male	85,276 (36.11)	150,910 (63.89)	*P* < 0.0001
	Female	16,149 (38.16)	26,167 (61.84)	
Wealth index	Poorest	11,578 (21.20)	43,034 (78.80)	*P* < 0.0001
	Poorer	15,154 (26.79)	41,405 (73.21)	
	Middle	18,456 (33.36)	36,863 (66.64)	
	Richer	23,566 (41.93)	32,635 (58.07)	
	Richest	32,672	23,142	
Covered by health insurance	No	90,799 (34.99)	168,731 (65.01)	*P* < 0.0001
	Yes	10,626 ( 56.01)	8346 (43.99)	
Husband educational level	No education	29,225 (28.71)	72,559 (71.29)	*P* < 0.0001
	Primary	24,323 (31.93)	51,860 (68.07)	
	Secondary	34,085 (43.73)	43,855 (56.27)	
	Higher	13,711 (61.19)	8696 (38.81)	
Currently working	No	34,954 (36.26)	61,456 (63.74)	*P* < 0.0001
	Yes	66,447 (36.52)	115,504 (63.48)	
Mass media exposure	No	35,610 (31.85)	76,207 (68.15)	*P* < 0.0001
	Yes	65,815 (39.48)	100,870 (60.52)	
Parity	Null parity	8048 (41.63)	11,284 (58.37)	*P* < 0.0001
	Multi	64,873 (39.40)	99,760 (60.60)	
	Grand	28,504 (30.15)	66,034 (69.85)	
Ownership of asset	Had not	80,618 (37.84)	132,412 (62.16)	*P* < 0.0001
	Had	20,807 (31.78)	44,665 (68.22)	
Involvement on decision making	Not involved	55,340 (33.50)	109,839 (66.50)	*P* < 0.0001
	Involved	46,085 (40.67)	67,239 (59.33)	

### Socioeconomic related inequality in barriers for accessing health care

**Fig. 1:**
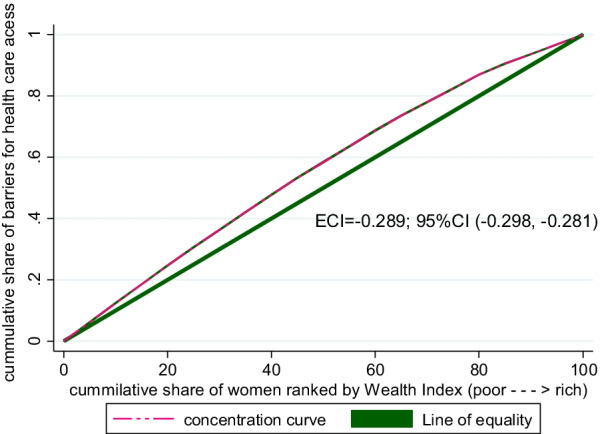
Concentration curve for barriers in accessing health care in Sub-Saharan Africa

The weighted Erreygers normalized concentration index (ECI) for barriers in accessing health care was − 0.289 with Standard error = 0.005 (*P* value < 0.0001) (Fig. 1). This revealed that a barrier in accessing health care was disproportionately concentrated among the poor (pro-poor). Similarly, the study showed that the concentration curve laying above the line of perfect equality which indicated a pro-poor inequality meaning barriers for accessing health care was disproportionately concentrated amongst married women from poorer households.

### Decomposing the socioeconomic related inequality in barriers for accessing health care

The decomposition analysis shows the contributions of individual variables to the overall socioeconomic inequality of barriers for accessing health care. To understand the factors that contribute to socio-economic inequality, coefficient and its significant level, elasticity, concentration index, and percent contribution were calculated.

Elasticity measures the change in the socioeconomic inequality in barriers for accessing in health care associated with a one-unit change in the independent variables [38, 39]. It has a positive and negative sign that indicates an increasing or decreasing change barrier for health care access in association with a positive change in the independent variables. For example, the elasticity for rural dwellers was 0.1725 which means, a 1% change in place of residence from urban to rural area will caused 17.25% increment in socioeconomic inequality of barriers for accessing health care. Whereas, a 1% change in the region of women’s educational level from no formal education to primary education level will result in − 6.12 changes (decrement) in socioeconomic inequality of barriers for accessing health care.

In addition, concentration index of the independent variables was computed that represents the distribution of the explanatory variables in reference to wealth quintiles. Like that of Elasticity, its sign can be positive or negative. A negative value of CI indicates that the variables of inequality were concentrated among poor households and a positive value indicates that the variables of inequality were concentrated among rich households. For instance, the study revealed that women from west Africa, aged from 20–24 and 45–49, rural area, primary education, whose husband had primary education, grand multi-parous and having ownership on their assets were more likely to be concentrated in the lower tail of the wealth distribution.

Moreover, percentage contribution was also estimated. It represents the relative contribution of each factor included in the analysis to the overall socioeconomic-related inequality in barriers for accessing health care. As the previous estimates, its value can be positive or negative where positive percentage contribution indicates a particular determinant that results in increasing the observed socioeconomic inequality in barriers for accessing health care and a negative percentage contribution indicates the one that results in decreasing the observed socioeconomic inequality in health variable (i.e. barriers for accessing health care). This study illustrated that the socioeconomic inequality in barriers for accessing health care was largely by the wealth variable (42.58%) followed by residence, were responsible for 36.42% of the socioeconomic inequality (Table 3).

**Table 3:** Contributing factors of socio-economic inequality in barriers for accessing health care in Sub-Saharan Africa

Variables	Category	Coefficient	Elasticity	CI	Absolute contribution	Percentage contribution
Regions of SSA	Central					
	East	− 0.1884*	− 0.2318	0.0153	− 0.0036	1.23
	North	− 0.3159*	− 0.0344	0.0088	− 0.0003	0.10
	West	− 0.2052*	− 0.3780	− 0.0215	0.0081	− 2.81
Subtotal					− 0.0282	1.68
Age	15–19					
	20–24	0.0231*	0.0196	− 0.0303	− 0.0006	0.21
	25–29	0.0228*	0.0266	0.0209	0.0006	− 0.19
	30–34	0.0214*	0.0235	0.0290	0.0007	− 0.24
	35–39	0.0221*	0.0208	0.0199	0.0004	− 0.14
	40–44	0.0252*	0.0153	0.0060	0.0001	− 0.03
	45–49	0.0289*	0.0136	− 0.0020	− 0.0001	0.01
Subtotal					0.0017	0.59
Residence	Urban					
	Rural	0.0732*	0.1725	− 0.6107	− 0.1054	36.42
Educational level	No education					
	Primary	− 0.0411*	− 0.0404	− 0.0612	0.0025	− 0.86
	Secondary	− 0.0830*	− 0.0645	0.2868	− 0.0185	6.39
	Higher	− 0.1530*	− 0.0209	0.1115	− 0.0023	0.81
Subtotal					− 0.0183	6.34
Household head sex	Male					
	Female	0.0063*	0.0037	0.0216	0.0001	− 0.03
Wealth index	Poorest					
	Poorer	− 0.0572*	− 0.0392	− 0.3288	0.0129	− 4.45
	Middle	− 0.1079*	− 0.0703	− 0.0024	0.0002	− 0.059
	Richer	− 0.1501*	− 0.1026	0.3208	− 0.0329	11.38
	Richest	− 0.2312*	− 0.1612	0.6410	− 0.1033	35.71
Subtotal						42.58
Covered by health insurance	No					
	Yes	− 0.0775*	− 0.0235	0.0909	− 0.0021	0.71
Husband educational level	No education					
	Primary	− 0.0113*	− 0.0097	− 0.0953	0.0009	− 0.32
	Secondary	− 0.0584*	− 0.0595	0.2149	− 0.0128	4.42
	Higher	− 0.1159*	− 0.0305	0.1789	− 0.0055	1.88
Subtotal					− 0.0174	5.98
Currently working	No					
	Yes	0.0133*	0.0084	0.0163	0.0001	− 0.05
Mass media exposure	No					
	Yes	− 0.0214*	− 0.0499	0.1782	− 0.0089	3.07
Parity	Null parity					
	Multi	0.0073	0.0075	0.1432	0.0011	− 0.37
	Grand	0.0190*	0.0187	− 0.1658	− 0.0031	1.07
Subtotal					− 0.002	0.70
Ownership of asset	Had not					
	Had	0.0251	0.0137	− 0.1161	− 0.0016	0.55
Involvement on decision making	Not involved					
	Involved	− 0.0312*	− 0.0367	0.1251	− 0.0046	1.59

^*^ = *P* value < 0.05

## Discussion

This study aimed to assess the socioeconomic inequality in barriers for accessing health care and its contributors among married reproductive aged women in sub-Saharan Africa. According to this study, the barrier for accessing health care was in favor of women from poor households. It is disproportionately concentrated among the poor community. Evidence has also supported that access to health care and economic class had strong relationships where limited access for health care is more prominent among the poor than rich community [40, 41]. This could have implied that socioeconomically disadvantaged women had limited access for maternal and child health services which had a great impact on women’s ability to enjoy a healthy life. Therefore, there is a need for an intervention among socioeconomically disadvantaged women to achieve the SDGs where the ultimate target is healthy lives for all at all ages.

In decomposition analysis, several factors were contributing for the pro-poor socioeconomic inequalities in barriers for accessing health care where wealth index, place of residency, husband educational level, women educational level, and mass media exposure were the major contributors for this inequality.

It was found that wealth quintiles were the major and important contributor for the overall socioeconomic inequality in barriers for accessing health care (42.58%). Previous studies had also documented that wealth is an important factor for health care access [42, 43]. This could be linked that poverty is intertwined with health in terms of accessibility of the facilities [44]. Even if it is not the case, it has a great effect on the utilization of the health facilities and health cares [45].

Following wealth index, residence was also the significant contributor for the overall socioeconomic inequality in barriers for accessing health care (36.42%). This finding was in line with previous literatures [46, 47]. This could be explained by women’s in rural areas had relatively poor healthcare-seeking behavior, had limited accessibility, and availability of health facilities [48, 49]. Rural residency might also imposes an extra cost for transportation as well as lack of availability of transportation and therefore they fail to attain the health facility to utilize health service [50]. As a result, women who have from rural area may become less motivated to seek care compared with their counterparts. Besides, women residing in rural areas have limited access for education and low chance of getting health information than women residing in urban areas [51, 52].

With respect to previous studies [53, 54], husband’s educational level was also another important contributor for socio-economic inequality for barriers in accessing health care. This could be linked with the fact that the involvement of husband on the health of their spouse is high if they had good knowledge on maternal health services which is highly interlinked with educational level [55] and good knowledge on maternal health services facilitates husbands positive participation and interest in their spousal health [56].

This study also revealed that women educational level was another contributor for the socio-economic inequality in barriers for accessing health care. Previous studies are also highlighted that educational attainment and utilization of health care had strong positive relationships [57–59]. The possible reason for this finding could be educational attainment is one markers of economic resources which enable women to take control of their own health and facilitate easy access to health care [60].

Regarding mass media exposure, it had significantly contributed for socioeconomic inequality for barriers in accessing health care. It is in agreement with the study done at south Asia [61]. This might be due to mass media is an important means of disseminating information concerning health and health care that may increases knowledge, attitude and practice of women towards health service utilization [62].

According to the findings of this study, program planners and decision makers should consider targeted interventions to minimize socioeconomic inequality for barriers in accessing health care such has increasing access to education to enhance women education level and their partner’s, enabling women to earn money, and increasing the coverage of mass media.

The findings of this study should be interpreted in light of the following limitations. First, due to cross-section nature of the data the findings cannot provide information on temporal relationships among the variables as a result casual inference couldn’t be drawn. Second, the study used wealth quintile as a measure of socioeconomic status but it didn’t use standardized living status measurement. If we use standardized living status measurement the wealth distribution in the upper tail such as middle, richer and richest wealth quintiles might fail below the line of poverty.

## Conclusion

There was a pro-poor inequality for barriers in accessing health care in sub-Saharan Africa. Wealth index, place of residency, husband educational level, women educational level, and mass media exposure were the major contributors for pro-poor socioeconomic inequalities barriers in accessing health care. Therefore, targeting disadvantaged women by interventions such as enabling women to make money and utilizing mass media to create awareness and enhance women empowerment will be helpful to alleviate these inequalities and achieve universal health coverage.

## Data Availability

Data cannot be shared publicly because of we use third parties DHS data set. Data are available from the measure DHS website/www.dhsprogram.com for researchers who meet the criteria for access to confidential data.

## Abbreviations

CI: Concentration Index
DHS: Demographic and Health Survey
ECI: Erreygers Concentration Index
DHS: Demographic and Health Survey
SSA: Sub-Saharan Africa
SDG: Sustainable Development Goal
WHO: World Health Organization
